# Identification by Mass Spectrometry and Immune Response Analysis of Guinea Pig Cytomegalovirus (GPCMV) Pentameric Complex Proteins GP129, 131 and 133

**DOI:** 10.3390/v6020727

**Published:** 2014-02-13

**Authors:** Josephine S. Gnanandarajah, Peter A. Gillis, Nelmary Hernandez-Alvarado, LeeAnn Higgins, Todd W. Markowski, Heungsup Sung, Sheila Lumley, Mark R. Schleiss

**Affiliations:** 1 Departments of Pediatrics, University of Minnesota Medical School, 2001 6th Street SE, Minneapolis, MN 55455, USA; E-Mails: gnan0007@umn.edu (J.S.G.); gill0221@umn.edu (P.A.G.); hernande@umn.edu (N.H.-A.); sung@amc.seoul.kr (H.S.); sheilalumley@gmail.com (S.L.); 2 Department of Biochemistry, Molecular Biology and Biophysics, University of Minnesota, 321 Church Street SE, Minneapolis, MN 55455, USA; E-Mails: higgi022@umn.edu (L.H.); marko025@umn.edu (T.W.M.)

**Keywords:** guinea pig cytomegalovirus, cytomegalovirus vaccine, pentameric complex, congenital cytomegalovirus infection, baculovirus

## Abstract

Development of a vaccine against congenital infection with human cytomegalovirus (HCMV) is a major public health priority. A potential vaccine target receiving considerable recent attention is the pentameric complex (PC) of HCMV proteins consisting of gL, gH, UL128, UL130, and UL131, since some antibodies against these target proteins are capable of potently neutralizing virus at epithelial and endothelial cell surfaces. Recently, homologous proteins have been described for guinea pig cytomegalovirus (GPCMV), consisting of gH, gL, and the GPCMV proteins GP129, GP131, and GP133. To investigate these proteins as potential vaccine targets, expression of GP129-GP133 transcripts was confirmed by reverse-transcriptase PCR. Mass spectrometry combined with western blot assays demonstrated the presence of GP129, GP131, and GP133 proteins in virus particles. Recombinant proteins corresponding to these PC proteins were generated in baculovirus, and as GST fusion proteins. Recombinant proteins were noted to be immunoreactive with convalescent sera from infected animals, suggesting that these proteins are recognized in the humoral immune response to GPCMV infection. These analyses support the study of PC-based recombinant vaccines in the GPCMV congenital infection model.

## 1. Introduction

Development of a vaccine against human cytomegalovirus (HCMV) is a major public health priority [1]. Although a vaccine could be useful in several patient populations at risk for HCMV−associated disease, a vaccine that could prevent congenital infection and its attendant sequelae would be of particular value. HCMV infection elicits both cellular and humoral immune responses. The suggestion that passively transferred anti-HCMV antibody during pregnancy protects the fetus against infection and HCMV-induced injury [2] has driven efforts to develop recombinant vaccines targeting major envelope glycoproteins. Development of an optimal glycoprotein vaccine is complicated by the complexity of virus entry and the variety of mechanisms employed. HCMV uses two different entry mechanisms to infect various cell types (fibroblasts, epithelial cells, endothelial cells, and macrophages). Fibroblast entry is mediated by glycoprotein complexes gB, gH/gL/gO, and gM/gN, but entry into epithelial cells, endothelial cells, and macrophages requires the gH/gL/UL128/UL130/UL131 complex [3,4,5]. Recently, it was demonstrated that the majority of virus−neutralizing antibodies in hyperimmune globulin target the gH/gL/UL128/UL130/UL131 complex [6]. Moreover, it was recently shown that fetal CMV transmission was more likely in the setting of a delayed or diminished maternal antibody response to the gH/gL/pUL128/130/131 complex during primary infection [7], further underscoring the potential usefulness of targeting these proteins for vaccine development [8]. Therefore, although clinical trials of recombinant gB vaccines (which have shown some degree of effectiveness in preventing CMV infection and disease in high risk populations) are ongoing [9,10], there is also considerable interest in developing vaccines targeting the HCMV pentameric complex (PC).

Ideally, a HCMV vaccine strategy would be tested in an animal model prior to clinical trials. Unfortunately, the strict species-specificity of CMVs precludes preclinical testing of HCMV vaccines in animals. However, a number of rodent and primate CMVs are useful in modeling HCMV vaccines and therapies, given the conservation of many immunogenic structural proteins amongst the various viruses [11,12,13]. The rhesus CMV (RhCMV) encodes highly conserved homologs of the UL128/UL130/UL131 members of the PC. Recently, a modified vaccinia virus Ankara virus (MVA) was described that stably coexpresses all five RhCMV proteins homologous to the HCMV PC [14]. RhCMV-naïve rhesus macaques vaccinated with the MVA construct developed antibodies that blocked infection of monkey kidney epithelial cells and rhesus fibroblasts. In addition, following subcutaneous RhCMV challenge at eight weeks post-vaccination, vaccinated animals demonstrated reduced plasma viral loads. 

Although the RhCMV model is a valuable system that recapitulates many of the pathologies of HCMV congenital infection, the expense of rhesus macaques and the difficulty in establishing RhCMV seronegative animal colonies makes it difficult to conduct large-scale studies comparing vaccines against congenital infection. Among the small animal models, the guinea pig CMV (GPCMV) is uniquely useful, since, in contrast to rodent models, transplacental infection of the fetus occurs following viral challenge during pregnancy [12,15]. Hence, the GPCMV model is well-suited to the study of vaccines against congenital infection. Inoue described a region of the GPCMV genome that appeared to be genetically unstable, in the setting of serial virus passage in fibroblasts that contained homologs of the HCMV UL128/UL130/UL131 genes [16,17]. More recently, Feierbach [18] further defined the proteins encoded by this region and determined that the GPCMV genes GP129, GP131, and GP133 represented the ancillary protein members (along with GP75 [gH] and gp115 [gL]) of the PC (Table 1). These studies were undertaken to characterize these genes and express the encoded proteins in recombinant systems, toward the goal of examining antibody responses of naturally and experimentally infected guinea pigs to these proteins. Knowledge of the patterns of immune responses to these GPCMV proteins should prove useful in the evaluation of potential subunit vaccines targeting the PC in the GPCMV model.

**Table 1:** Guinea pig cytomegalovirus (GPCMV) 129-133 Locus.

GPCMV Protein	HCMV Homolog	Codons	Predicted M_r_	Amino Acid Similarity to HCMV (%)
GP129	UL128	179	20.6 kDa	48%
GP131	UL130	192	21.8 kDa	36%
GP133	UL131	127	14.7 kDa	29%

## 2. Results and Discussion

### 2.1. Transcriptional Analyses of GP129, 131 and 133

**Figure 1:**
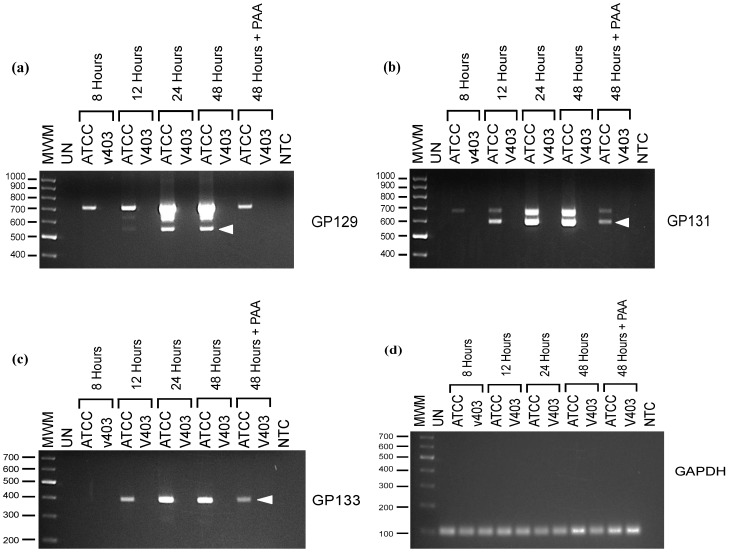
Transcription of GP129, 131 and 133 as assessed by reverse-transcriptase PCR (RT-PCR). RNA from ATCC GPCMV-infected cells (Lanes 2, 4, 6, 8, 10) and vAM403-infected cells (Lanes 3, 5, 7, 9 and 11) was purified at 8 hours (Lanes 2, 3), 12 hours (Lanes 4, 5), 24 hours (Lanes 6, 7) and 48 hours (Lanes 8, 9) post-infection. A 48-hour time point was also collected in the presence of phosphonoacetic acid (PAA) (Lanes 10, 11). Lane 1 represents RT-PCR of RNA from uninfected cells. Electrophoresis was performed on a 1.5% agarose gel. Molecular weights markers are the 1 Kb-Opti-DNA marker from ABM (Applied Biological Materials Inc., Richmond, BC, Canada). (**a**) GP129-specific transcripts. Predicted splice product of 540 nt is noted (arrow), but is not present in infected cells incubated with PAA. (**b**) GP131-specific transcripts. Predicted splice product of 515 nt is noted (arrow). (**c**) GP133-specific transcripts demonstrating no evidence of splicing. Predicted 384 nt product is noted (arrow). (**d**) GAPDH control.

Initially, transcription from the GPCMV GP129, 131, and 133 locus was examined by reverse−transcriptase PCR (RT-PCR). Primer design was based upon published primer sequences reported by Inoue [17]. Primers used for these studies are summarized in Table 2. Previous reports indicated that these genes are encoded by single mRNAs, and the GP129 and GP131 RNAs, but not the GP133 RNA, are spliced (Figure 1A). To confirm and extend these findings, RNA was purified from GPCMV-infected GPL cells at 8, 12, 24, and 48 hours post-inoculation. A 48-hour time point was also collected in the presence of phosphonoacetic acid (PAA), 300 µg/mL. The viral stock used was P1-passaged GPCMV Strain 22122 purchased directly from the American Type Culture Collection (ATCC; Manassas, VA, USA). As an additional control, cells were inoculated with vAM403, an eGFP-expressing recombinant GPCMV [19] known to lack the 1.6 kb region containing the GP129-133 locus [16]. The RT-PCR products predicted for each gene were: 540 base pairs (bp) for GP129 transcript; 515 bp for GP131; and 384 bp for GP133. Transcripts specific for GP131 and GP133 were apparent as early as 12 hours post-infection (Figure 1B,C). A GP129-specific transcript could be faintly detected as soon as 12 hours post-infection, and was abundantly present at 24 hours post−infection (Figure 1A). These data differ from those reported by Inoue and colleagues [17], who did not note any GP129-specific transcript until ~48 hours post−infection, although this analysis was performed by northern blot, which may have been less sensitive than RT-PCR. Similar to Inoue’s findings, we noted that the spliced GP129 transcript was not identified in the presence of PAA, consistent with its classification as a late gene. However, we noted transcripts corresponding to the spliced GP131 mRNA as soon as 12 hours post-infection and continuing at high levels throughout 48 hours post-infection even in the presence of PAA (Figure 1B), suggesting that GP131 is an early gene, in contrast to the previous report by Inoue that suggested (based on northern blot analysis) that both GP129 and GP131 were late genes [17]. 

**Table 2:** Primers used in GP129, 131 and 133 Cloning and Protein Expression Studies.

ORF	Primer Sequences
GP129 (Baculovirus and Transcript Analysis)	5' – TACG*CTGCAG*AATGCGTGTTATTGTT – 3' (F)
	5' – TACG*AGATCT*TTACTTCCCGTTACC – 3' (R)
GP129 GST (R1)	5' – TACG*GGATCC*TATACCCGTCCCGGTATCTTTG – 3' (F)
	5' – TACG*CTCGAG*TTAAGTATTCCCACATCGTACTAATC – 3' (R)
GP129 GST (R2)	5' – TACG*GGATCC*TCGCGGCAAGAACTCCAT – 3' (F)
	5' – TACG*CTCGAG*TTAACGGTAGGTCACCCCCAAG – 3' (R)
GP129 GST (R3)	5' – TACG*GGATCC*AACGGTTTATTATGCACCTTTC – 3' (F)
	5' – TACG*CTCGAG*TTACTTCCCGTTACCATCGAC – 3' (R)
GP131 (Baculovirus)	5' – TAC*GAATTC*ATGATGAAACGATAT – 3' (F)
	5' – TACG*CTGCAG*TTATCACGTCCAGTT – 3' (R)
GP131 (Transcript Analysis)	5' – ATAATGATGAAACGATAT – 3' (F)
	5' – TTATCACGTCCAGTTCCA – 3' (R)
GP131 GST (R1)	5' – GATA*GGATCC*TTTTACGCCTCGTTCGGA – 3' (F)
	5' – AATA*CTCGAG*GTTCGTCAGGGTCAGGAC – 3' (R)
GP131 GST (R2)	5' – GATA*GGATCC*CGCCGAATAGATTACGGA – 3' (F)
	5' – AATA*CTCGAG*CCACAAGAAGGACGAATC – 3' (R)
GP131 GST (R3)	5' – CGTG*GGATCC*TGGCATTATACGATACGG – 3' (F)
	5' – AATA*CTCGAG*CAGGCAAGCGATAGAATC – 3' (R)
GP133 (Baculovirus)	5' – TACG*CTGCAG*TATGTTTTGGCGTCTTGTA – 3' (F)
	5' – TACG*AGATCT*TTATGCTCTGTCTATGC – 3' (R)
GP133 (Transcript Analysis)	5' – TATGTTTTGGCGTCTTGTA – 3' (F)
	5' – TTATGCTCTGTCTATGC – 3' (R)
GP133 GST (R1)	5' – GATC*GGATCC*ACAAGAGTTAAGAAAGAAAACCAACTG – 3' (F)
	5' – GATC*CTCGAG*TTAGGAGTCCGCTAACGTATG – 3' (R)
GP133 GST (R2)	5' – GATC*GGATCC*AGGGGACGTTACAGGAAAGG – 3' (F)
	5' – GATC*CTCGAG*TTAAGTGCTTTGTTGAATAGAAATACG – 3' (R)
GAPDH	5' – ATCTCATCGTATTTGGCCGGT – 3' (F)
	5' – AATGGGAAGCTCACAGGTATGG – 3' (R)

We saw no evidence of splicing of the GP133 transcript, and this RNA continued to be expressed to high levels through 48 hours post-infection. This transcript was also expressed in the presence of PAA. RNA species larger than we predicted were noted for the GP129 and GP131 transcripts. Absence of any signal in the absence of reverse transcriptase makes the possibility of DNA contamination unlikely. These were presumed to represent larger, unspliced RNAs driven by promoters upstream of the GP129-133 promoter region. RNA purified from vAM403-infected cells was negative for GP129, 131 and 133 RT-PCR products, as expected, since this virus lacks the region of the GPCMV genome encoding these ORFs. Guinea pig GAPDH cDNA was amplified to control for RNA recovery (1D). No bands were noted in the no-RT control (data not shown).

### 2.2. Mass Spectrometry Analysis

**Figure 2:**
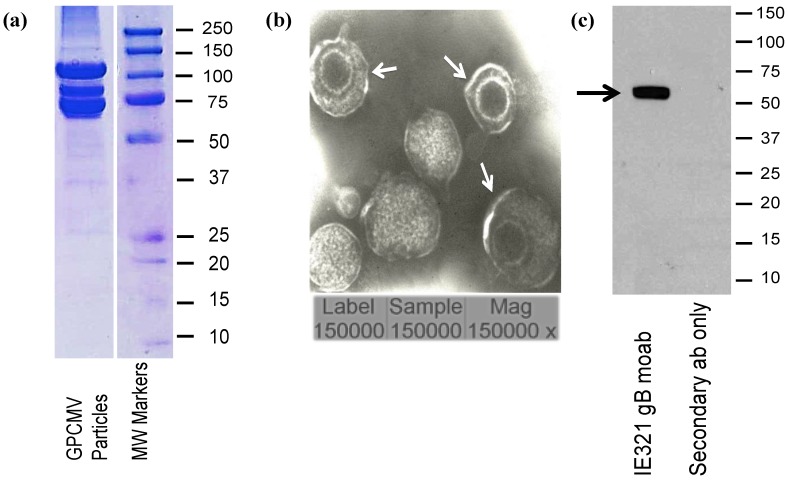
Characterization of GPCMV viral particles. (**a**) Coomassie blue stain (5 µg) of purified virus particles by 12% gel SDS-PAGE. (**b**) Transmission electron microscope microphotograph of purified particles. Negative contrast preparation of purified particles demonstrates enveloped virions (arrows). Dense bodies are also noted. Magnification, 150,000×. **(c)** Western blot analysis of viral particles probed with IE3-21 monoclonal antibody recognizing GPCMV gB (1:2,000 dilution; arrow).

**Appendix Figure A1:**
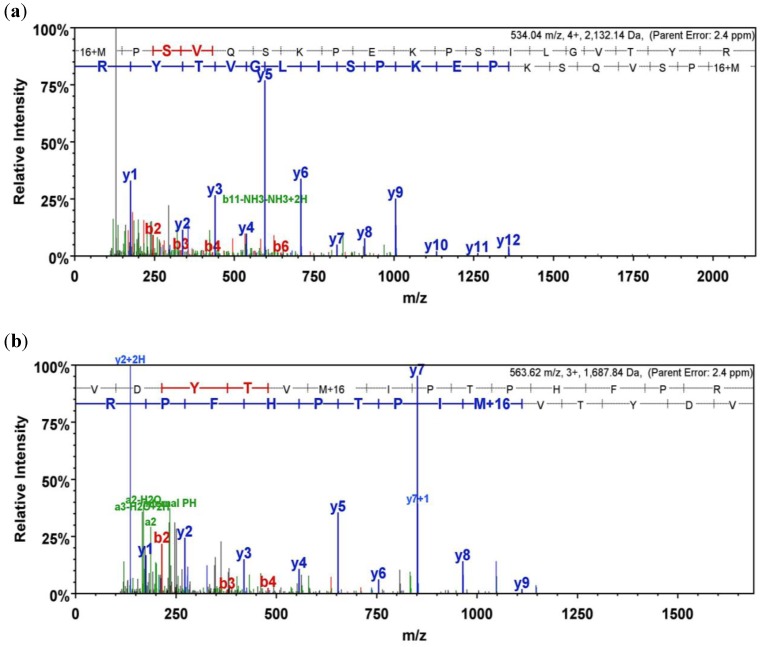
Mass spectrometry data for GP129. Representative mass spectra for each of the unique peptides identified using Velos Orbitrap LC-MS/MS system for GP129. B peaks are labeled in red. Y peaks are labeled in blue. Additional charge variants are marked in green. (**a**) Spectrum for MPSVQSKPEKPSILGVTYR. (**b**) Spectrum for VDYTVMIPTPHFPR.

**Appendix Figure A2:**
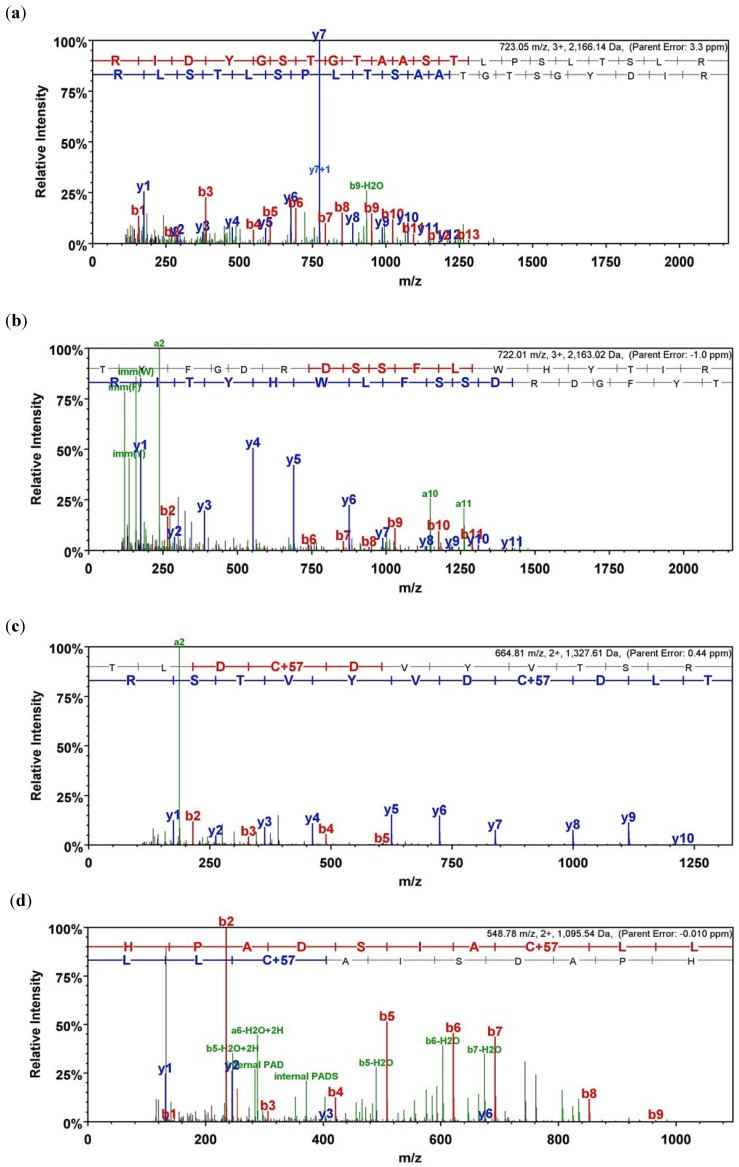
Mass spectrometry data for GP131. Representative mass spectra for each of the unique peptides identified using Velos Orbitrap LC-MS/MS system for GP131. B peaks are labeled in red. Y peaks are labeled in blue. Additional charge variants are marked in green. (**a**) Spectrum for RIDYGSTGTAASTLPSLTSLR. (**b**) Spectrum for TYFGDRDSSFLWHYTIR. (**c**) Spectrum for TLDCDVYVTSR. (**d**) Spectrum for HPADSIACLL.

**Appendix Figure A3:**
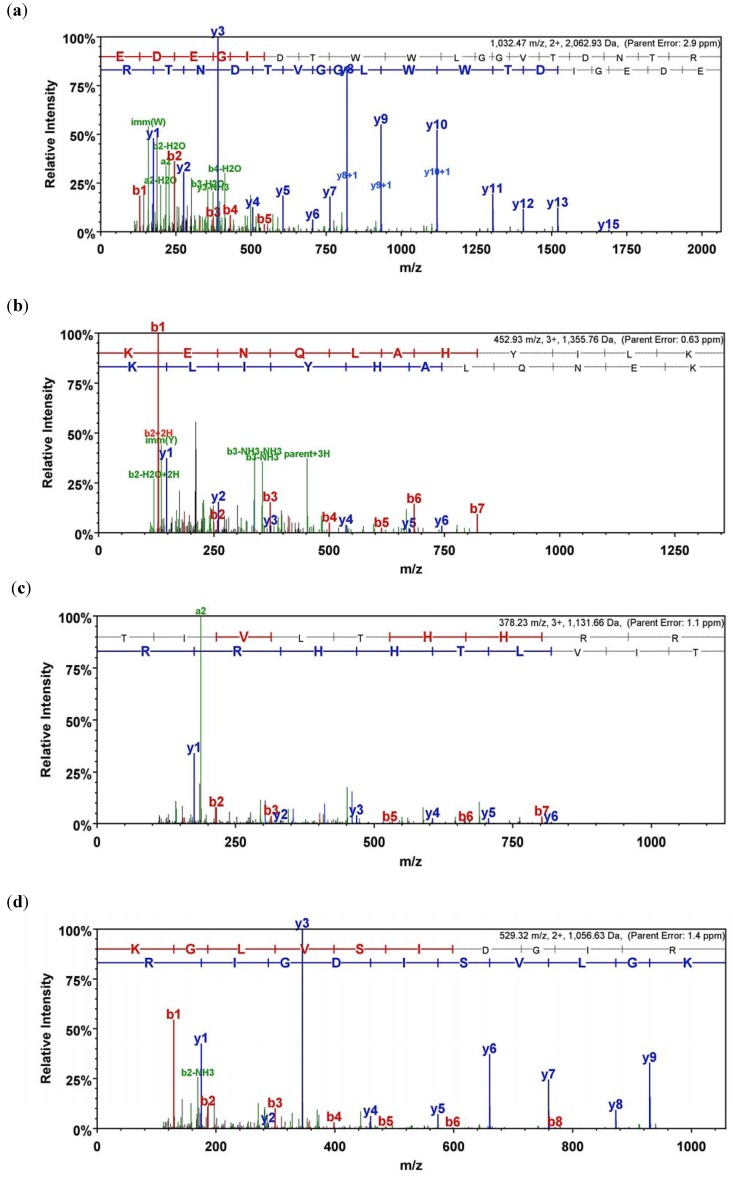
Mass spectrometry data for GP133. Representative mass spectra for each of the unique peptides identified using Velos Orbitrap LC-MS/MS system for GP133. B peaks are labeled in red. Y peaks are labeled in blue. Additional charge variants are marked in green. (**a**) Spectrum for EDEGIDTWWLGGVTDNTR. (**b**) Spectrum for KENQLAHYILK. (**c**) Spectrum for TIVLTHHR. **(d**) Spectrum for KGLVSIDGIR.

GPCMV virus particles were purified by sequential sucrose [20] and glycerol-tartrate density gradient centrifugation [21] as described in Section 3.2. Particles were examined by coomassie blue stain (Figure 2a) and transmission electron microscopy (EM; Figure 2b) to confirm homogeneity. Particles were probed with a monoclonal antibody (moab) to glycoprotein B, IE321, generated in our laboratory (Figure 2c), to further confirm their integrity. Particles were resolved by SDS PAGE followed by coomassie staining, and bands were excised from the gel for mass spectrometry. Three peptides corresponding to two unique sequences for GP129 were identified from a region of the gel with an approximate molecular weight of 25 kD. Additionally, six peptides corresponding to four unique sequences corresponding to GP131 were identified from a protein band excised from a region with a molecular weight spanning approximately 20–22 kD. Finally, seven peptides from four unique sequences of GP133 were identified from a protein band with an approximate molecular weight of 18 kD. Results are summarized in Table 3. Mass spectra data are provided in Appendix Figures A1–A3. 

**Table 3:** Mass spectrometry identification of peptides corresponding to GP129, 131 and 133. Other major virion structural proteins included as controls for mass spectrometry analyses (protein probability calculations performed as described in materials and methods).

ORF	Unique Spectra	Unique Peptide Sequences	% Coverage	Max X_corr_	Probability
GP 129	2	MPSVQSKPEKPSILGVTYR VDYTVMIPTPHFPR	18	3.37	99%
GP131	6	RIDYGSTGTAASTLPSLTSLR TYFGDRDSSFLWHYTIRT DCDVYVTSR HPADSIACLL	29	5.01	99.9%
GP133	7	EDEGIDTWWLGGVTDNTR VKKENQLAHYILK KGLVSIDGIR TIVLTHHR	39	4.51	99.9%
GP83	74	61	59	62	100%
GP25	78	55	77	5.9	100%
gB	51	37	31	5.1	100%

### 2.3. Characterization of GP129 and 131 Proteins

**Figure 3:**
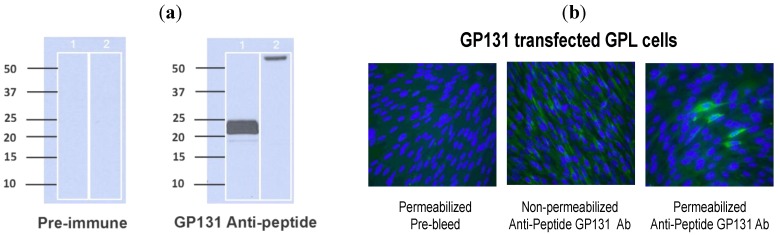
Characterization of GP131 protein. (**a**) Western blot analysis using anti-peptide GP131 antibody. Virion particles were probed with either pre-immune sera (left panel) or immune sera (right panel) following purification of virus particles from cells infected either with ATCC GPCMV (Lane 1 in each panel) or vAM403 (Lane 2 in each panel). A band of approximately 20–22 kDa was noted with immune, but not pre-immune sera. On shorter exposure, this band appeared as a doublet. (**b**) Effect of cell permeabilization on detection of GP131. GPL cells were transfected with a GP131 expression plasmid and immunofluorescence performed with anti-GP131 antibody. Little to no protein expression was detected in non−permeabilized cells (middle panel), but protein expression was readily detected following permeabilization (right panel). Left panel demonstrates pre-immune antibody control.

Initially, an anti-peptide rabbit antiserum was generated targeting a GP131-specific peptide sequence, NH_2_-CYYPSTPIPKSFVKHVDTTRSLPE-COOH, conjugated with keyhole limpet hemocyanin (see Section 3 for details on generation of antibody). This antibody was used in western blot assay to examine purified virions (purified from passage 1 ATCC Strain 22122 GPCMV), as well as infected cell lysates, for expression of the GP131 protein. Immune sera consistently demonstrated the appearance of a broadly migrating band at approximately 22 kDa. In some experiments, the band appeared to separate into two discrete bands at ~20 and ~22 kDa (Figure 3A). To confirm the specificity of this response for GP131, westerns were also performed using a recombinant, eGFP-expressing GPCMV, vAM403, known to lack the 1.6 kb region spanning the GP129-133 locus. Western assay targeting vAM403 proteins using the GP131 rabbit polyclonal antibody did not demonstrate presence of this band in purified lysates from cells infected with this virus, as expected (Figure 3A, Lane 2).

The GP131-specific antibody was next examined in an immunofluorescence assay using cells transfected with a GP131 expression plasmid, pKTS 789. Immune, (but not pre-immune) sera identified a protein in GP131-transfected cells, but only under conditions of permeabilization (Figure 3B). This result is compatible with the known requirement of GP131 to be co-expressed with gH in order for cell surface localization.

**Figure 4:**
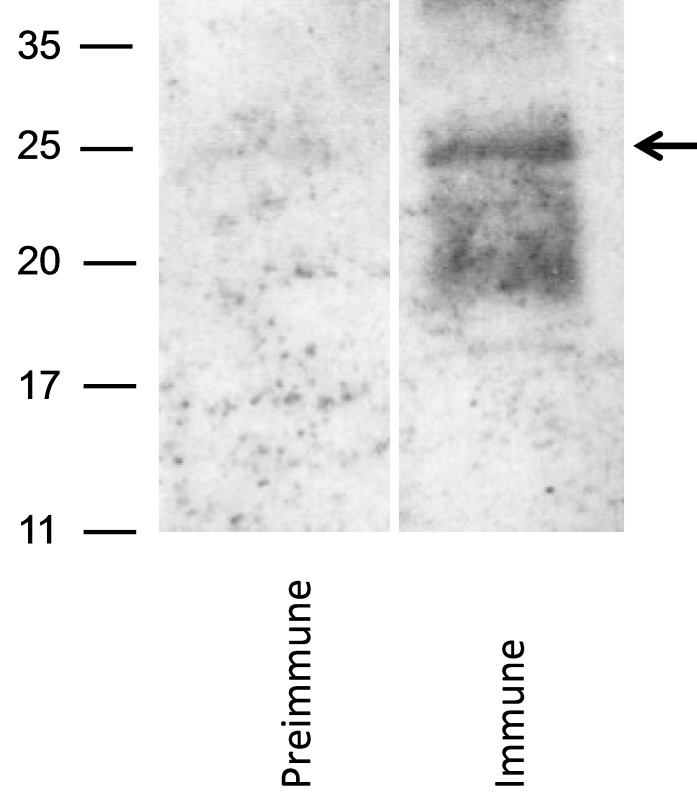
Identification of GP129 in virus particles. Western blot analysis using an anti-GST/GP129 fusion protein derived antibody. Following electrophoresis with 12% Bis-Tris gel, western transfer was performed and virion particles were probed (1:200 dilution) with either pre-immune sera (left panel) or immune sera (right panel). GST_R3_129 immune (but not pre-immune) antisera identified a broadly migrating band with MW of the predominate species at ~25 kDa (arrow). Band was not identified in blots of vAM403 (lacking GP129-133 locus) virus particles probed with identical antibodies (data not shown).

To identify GP129 in virions, a GP129-specific antibody was next generated following vaccination of naïve guinea pigs with a GST fusion protein, designated GST_R3_GP129 (described in Section 3). Three guinea pigs were immunized with fusion protein. All animals generated ELISA responses to GST_R3_129 (data not shown). The GST_R3_GP129 immune, but not pre-immune, sera identified a broadly migrating band in purified GPCMV virions (Figure 4). This result suggested that the GP129 protein is expressed in virions, and migrates broadly in the ~25 kDa range by SDS-PAGE. These data confirmed the identification of GP129-specific peptides present in the mass spectrometry analyses of virus particles.

### 2.4. Characterization of Guinea Pig Antibody Reactivity to Recombinant-Expressed GP129, 131, and 133

**Figure 5:**
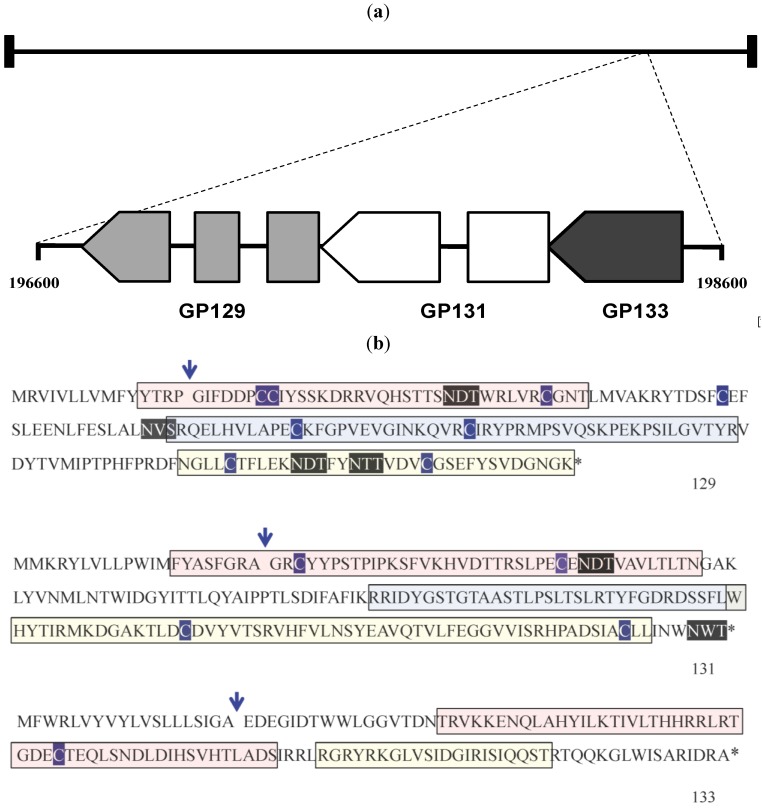
Subcloning of GP129, 131, 133 proteins. (**a**) Schematic map of the GPCMV GP129, 131 and 133 ORFs, patterned after [18]. These ORFs map to the rightward end of the GPCMV genome. Genome coordinates of this region are indicated (based on sequence Genbank KC503762 [24]). In contrast to HCMV, the GP131 (UL130) homolog is spliced, but the GP133 (UL131 homolog) is not. (**b**) GPCMV GP129, GP131, and GP133 ORFs. Predicted van Heinje signal sequence cleavage site is indicated by arrow. Cysteine residues are outlined in dark blue. Potential glycosylation sites (N-X-T and N-X-S) are shown in black outline. Regions of ORFs subcloned as GST fusion proteins are indicated. For GP129 and GP131, pink region represents R1 region; blue, R2 region; yellow, R3. For GP133, pink region is R1 and yellow region R2 (see Table 2 and text for details).

**Figure 6:**
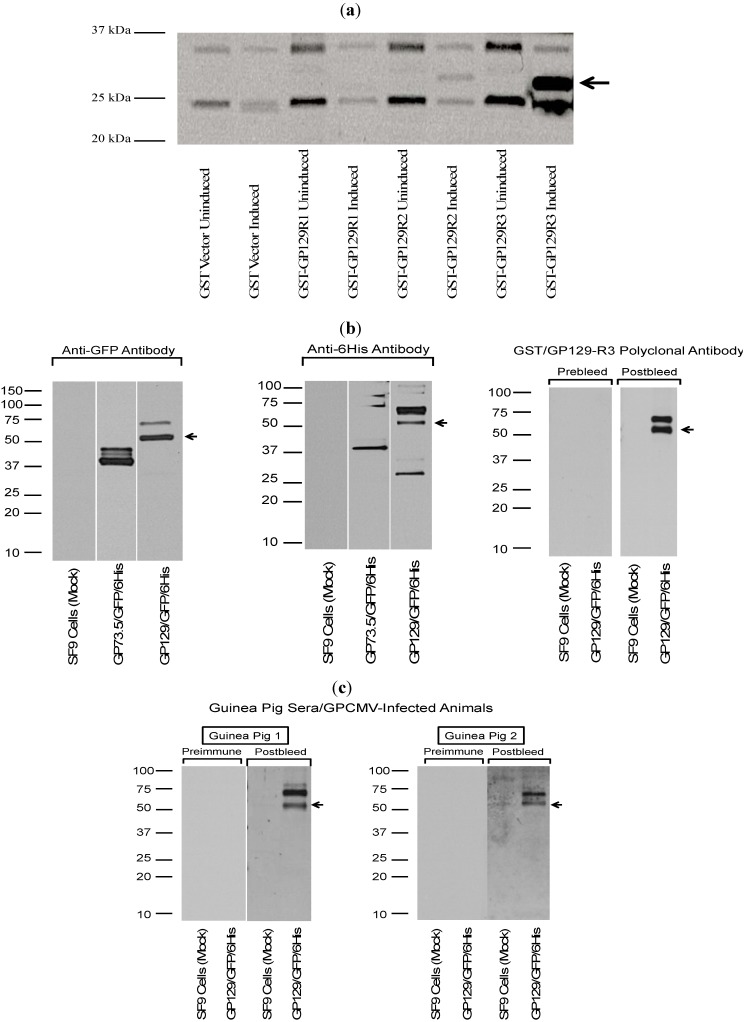
Antibodies from GPCMV-seropositive guinea pigs react with recombinant forms of GP129. (**a**) Analysis of GP129/GST fusion proteins. Three GP129 domains (Figure 5b) were cloned in-frame with GST using vector pGEX 6-P as described in materials and methods. Constructs evaluated by western blot included GST only; GST-GP129R1 fusion; GST-GP129R2 fusion; and GST-GP129R3 fusion, without IPTG induction (uninduced) and following IPTG induction (induced). Anti-GPCMV antisera from immune, experimentally infected guinea pigs demonstrated strongest immunoreactivity with GST−GP129R3 fusion protein. In some experiments, some signal could be observed with GP129R2 fusion protein. Data shown is a representative result from an experimentally infected animal. All fusion proteins expressed to high levels following IPTG induction as demonstrated by western analysis with anti-GST monoclonal antibody (data not shown). No background reactivity was noted with serum from GPCMV-seronegative animals or with pre-immune sera from experimentally infected guinea pigs (data not shown). (**b**) Western blot analysis of recombinant baculovirus-expressed GP129. The GP129 was cloned in-frame with GFP and a 6-His tag. This resulted in a fusion protein of ~55 kDa, made up of ~6 kDa from the 6-His tag and vector sequences; ~28 kDa from the GFP sequence; and ~21 kDa from the GP129 sequences. Purified recombinant protein was subjected to SDS-PAGE and western assays performed as described in materials and methods section. For some western blots, a control baculovirus expressing a recombinant form of the GPCMV GP73.5 glycoprotein was also evaluated. Top panel, both the anti−GFP and anti-6 His antibodies were immunoreactive with GP73.5 (~37 kDa) and GP129 (~55 kDa) fusion proteins, as expected. A higher MW band was noted at ~65–70 kDa, possibly representing a glycosylated variant of the baculovirus-expressed GP129 fusion protein. Sera, raised against the GST-GP129-R3 fusion protein (Figure 5a), was also used to probe baculovirus-expressed GP129 as a control. Immune, but not preimmune, sera (1:200 dilution) from a guinea pig immunized with the GST fusion protein were reactive with the baculovirus expressed protein, but not proteins in uninfected SF9 cells. Again, both the 55 kDa fusion protein as well as a larger species of 65–70 kDa, were identified. (**c**) Sera from experimentally infected animals were immunoreactive with the GP129−baulovirus fusion protein. Results shown are representative pre- and post-infection sera (1:200 dilution) from two GPCMV-infected guinea pigs (GP1; GP2) demonstrating immunoreactivity with bacolovirus-GP129 fusion protein (arrow), but not a pre-immune guinea pig serum (NEG).

To examine the guinea pig immune response to GP129, 131, and 133, a series of GST fusion proteins were generated, corresponding to predicted immunogenic domains in the respective proteins. Primers used in the generation of these fusion proteins are indicated in Table 2. Immune responses in guinea pig convalescent sera from infected animals were readily demonstrable to GP129 fusion proteins. Three domains of GP129 (GP129 R1, R2, and R3; Figure 5) were cloned as GST fusion proteins and western blots probed with anti-GPCMV antisera obtained from experimentally infected animals. The R3 domain (Figure 6A) was the domain most highly immunoreactive with convalescent anti-GPCMV sera from infected animals. This region of the GP129 contains a cysteine residue highly conserved with HCMV and RhCMV UL128 (the homolog of GP129). Although interactions between UL128 and the other constituents of the pentameric complex are not believe to involve disulfide bond formation, this region of the UL128 protein in HCMV is known to be the target of neutralizing monoclonal antibodies [22,23]. When GP129 was expressed in recombinant baculovirus, purified recombinant protein was also immunoreactive with immune sera from animals challenged with salivary gland-adapted GPCMV (Figure 6B). Pre-immune sera failed to recognize the recombinant protein. These analyses strongly suggest that the GP129 protein is a target of the guinea pig antibody response following GPCMV infection.

**Figure 7:**
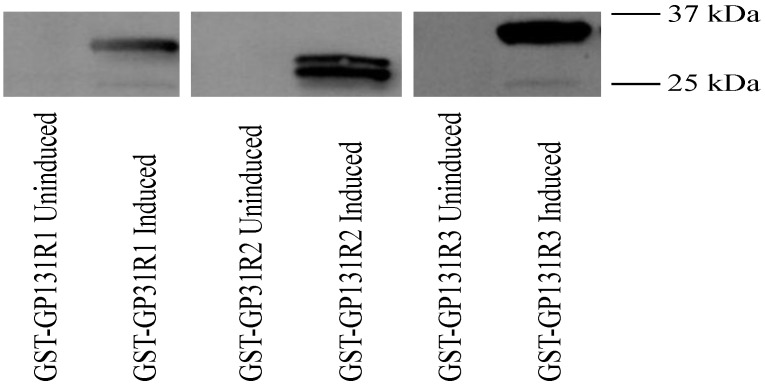
Characterization of antibody responses of GPCMV-infected guinea pigs to recombinant GP131 proteins. Western blots examining reactivity of anti-GPCMV antisera with uninduced and IPTG-induced GST-GP131R1, R2, and R3 fusion proteins. GP133R1 and GP131R3 were most consistently reactive with post-infection antisera (1:200 dilution), but not preimmune sera (data not shown). Representative result from an infected animal is shown. 37 and 25 kDa markers are indicated in right side of figure.

The guinea pig antibody response to GP131 was next evaluated, using recombinant GP131 in western blot assay along with sera from GPCMV-seropositive guinea pigs. As was the approach for GP129, the GP131 coding sequence was first subcloned as three GST fusion proteins, spanning potentially immunogenic domains of the GP131 protein (GP131 R1, R2 and R3). The GST fusion proteins were purified and, following western transfer, blots were probed with sera from experimentally infected GPCMV-seropositive animals (1:200 dilution; details as noted in Figure 6). A representative result from a GPCMV-infected animal is shown. These analyses demonstrated (Figure 7) that convalescent, but not pre-immune, sera from GPCMV-infected guinea pigs recognized the GST fusion protein corresponding to domains R1, R2 and R3 of the GP131 ORF. 

**Figure 8:**
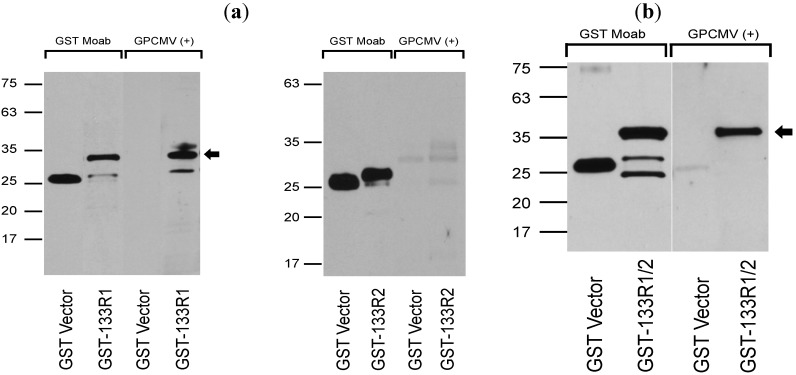
Characterization of antibody responses from GPCMV-immune guinea pigs to recombinant GP133 protein. (**a**) Antisera from a GPCMV-infected animal [GPCMV (+)] demonstrates immunoreactivity with the GST-GP133_R1_fusion protein (arrow) but not with purified GST protein. Sera is not reactive with GST-R2 fusion protein (right panel). Anti−GST moab (1:5,000 dilution) demonstrated reactivity both with the GST-GP133R1, R2, and R1/2 fusion proteins, as well as the purified GST protein. (**b**) Western analysis of GST−GP133R1/2 fusion protein. Both GST (GST vector) and GST-GP133R1/2 fusion protein are reactive with anti-GST moab; however, only sera from GPCMV-infected animal [GPCMV(+), 1:200 dilution] was reactive with the GST-R1/2 fusion protein. For both experiments, pre-immune sera from GPCMV-infected guinea pig was unreactive with all GP133/GST fusion proteins and with the GST parent protein itself (data not shown).

Finally, antibody responses to recombinant GP133 proteins were assessed. GP133 coding sequences were subcloned as depicted in Figure 5b. GST fusion constructs corresponding to Region 1 (predicted MW of 31.8 kDa) and Region 2 (predicted MW of 28.5 kDa) were evaluated, as well as a full-length GST fusion construct spanning both domains (R1/R2 construct; predicted MW of 34.8). These fusion protein clones were generated using primers as depicted in Table 2. The GST fusion proteins were purified from insoluble inclusion bodies and, following western transfer, blots were probed with convalescent sera from GPCMV seropositive animals that had been infected subcutaneously with SG virus. Results were confirmed with three independently GPCMV-infected guinea pigs; Figure 8 demonstrates representative results from one of these animals. These analyses demonstrated that convalescent, but not pre-immune (data not shown), sera from GPCMV-infected guinea pigs recognized the proteins corresponding to both the R1 and the R1/R2 regions of GP133 expressed as GST fusions, but not the R2 region of the GP133 protein. Reactivity of sera from infected animals, in contrast, was not noted with recombinant GP133 expressed as a GFP/6-His fusion protein in recombinant baculovirus (data not shown). This may have been due to loss of reactive epitopes engendered by fusion of the GFP and 6-His tags on the amino-terminal coding sequences of the recombinant GP133 protein. Further experiments will be necessary to resolve this question.

Table 4 summarizes the immunoreactivity of the various GST fusion constructs (depicted in Figure 5b) with immune sera from infected guinea pigs post-infection with SG virus. Designations: (−), no immunoreactivity; (+), minimal immunoreactivity; (++), moderate immunoreactivity; (+++) consistent, high-level immunoreactivity.

**Table 4:** Summary of reactivity of sera from infected guinea pigs with GST-GP129, 131 and 133 fusion proteins.

GST Fusion Protein Domain	Immunoreactivity with Guinea Pig Sera Post-Experimental Infection with GPCMV
GP129-R1	−
GP129-R2	+
GP129-R3	+++
GP131-R1	++
GP131-R2	+
GP131-R3	+++
GP133-R1	++
GP133-R2	−

## 3. Experimental Section

### 3.1. Cells and Virus

GPCMV (Strain 22122/VR682 from American Type Culture Collection [ATCC], Manassas, VA, USA), and vAM403, an enhanced green fluorescent protein (eGFP)-tagged GPCMV [19] were propagated on guinea pig fibroblast lung cells (GPL; ATCC CCL 158) in F-12 medium supplemented with 10% fetal calf serum (FCS; Gibco-BRL), 10,000 IU of penicillin/liter, 10 mg of streptomycin/liter, and 7.5% NaHCO3 (Gibco-BRL). 

### 3.2. EM and Mass Spectrometry Analyses

For mass spectrometry analyses, GPCMV (Strain 22122; ATCC VR-682) was propagated on GPL cells as described previously, and virus particles were purified from culture supernatants by density gradient centrifugation. Virus was purchased from ATCC and subjected to only one passage in GPL culture to minimize selection for genome variants with deletion in the GP129-133 locus [16]. Briefly, cellular debris was removed from the culture supernatant by repeated centrifugation at 4,000 × g for 15 min and supernatant was collected. GPCMV particles were separated by centrifugation at 11,000 × g for 30 min and pellet was washed twice in phosphate-buffered saline (PBS). Virion particles were resuspended in PBS and loaded onto a 20%–70% sucrose gradient (27 mL) in SW 32 centrifuge tubes and subjected to ultracentrifugation at 22,000 RPM/25 °C for 60 min. The visualized band was then subjected to glycerol-tartrate gradient centrifugation as described elsewhere [21]. Particles were washed and protein concentration was quantified by BCA assay (Thermo Fisher Scientific, Waltham, MA, USA). Particles were also subjected to electron microscopy. First, particles were centrifuged at 30 PSI using an airfuge (Belkman-Coulter, Brea, CA, USA) for 20 minutes on parafilm and formvar coated copper grids (Electron Microscopy Sciences, Hatfield, PA, USA). Excess liquid was wicked and the grids were stained with 1% phosphotungstic acid for one minute. All sections were observed under JEOL 1200 EX II transmission electron microscope (JEOL LTD, Tokyo, Japan). Images were obtained using a Veleta 2K × 2K camera with iTEM software (Olympus SIS, Munster, Germany) [25]. GPCMV particles were then resolved by SDS-PAGE and resolved protein bands were visualized by Coomassie stain. Visible bands were excised and subjected to in-gel trypsin digestion [26]. The digested peptide mixtures were analyzed using LTQ LC-MS/MS system or Velos Orbitrap LC-MS/MS system (ThermoFisher, San Jose, CA, USA) as previously described [27,28]. Tandem mass spectra were searched with Sequest version 2.7 (ThermoFisher, San Jose, CA, USA) and peptide and probability scores were applied in Scaffold 3.6.5 (Proteome Software, Inc., Portland, OR, USA) [29] as specified by Protein Prophet [30] and Peptide Prophet [31]. Mass spectra were searched against a combined protein FASTA database with sequence entries from NCBI Reference Sequence *Cavia porcellus* [32] NCBI nr *Caviid herpesvirus 2* [33] and common lab contaminants from [34]. Manual inspection of all spectra validated the peptide assignments made by Scaffold.

### 3.3. Transcriptional Analyses

Guinea pig lung fibroblasts (GPL) were infected with ATCC GPCMV P1 virus at MOI of 0.1. After one hour of infection at 37 °C, cells were washed with PBS and cultured in F12 medium supplemented with 10% fetal calf serum, 10,000 IU/L penicillin, 10 mg/L streptomycin, and 7.5% NaHCO3. RNA was extracted from infected GPL cells at 8, 12, 24 and 48 hours post-infection using RNeasy mini kit (Qiagen, Venlo, Limburg, NL) according to the manufacturer’s instructions. RNA was treated with RNase−free DNase Set (Qiagen) while in the column according to manufacturer’s instructions. cDNA was synthesized from 1 μg of total RNA using either Quantitect Reverse Transcription kit (Qiagen). Conventional PCR was carried out using cDNA as template and goTaq (Promega, Madison, WI, USA). The PCR for GAPDH were done using primer pair 5'-ATCTCATCGTATTTGGCCGGT-3' and 5'-AATGGGAAGCTCACAGGTATGG-3'. The conditions for GAPDH PCR were: initial denaturation at 95 °C for 2 min, followed by 95 °C for 30 s, 58 °C for 30 s, 72 °C for 35 s for a total of 25 cycles, and elongation at 72 °C for 7 min. GP129 PCR primers are listed in Table 2. The conditions of the GP129 PCR were: initial denaturation at 95 °C for 2 min, followed by 95 °C for 30 s, 56 °C for 30 s, 72 °C for 35s for a total of 35 cycles, and elongation at 72 °C for 7 min. The primers for GP131 PCR were 5'-ATAATGATGAAACGATAT-3' and 5'-TTATCACGTCCAGTTCCA-3'; the conditions were the same as GP131 but the annealing temperature was 48 °C. GP133 PCR was done using primer pair 5'-TATGTTTTGGCGTCTTGTA-3' and 5'-TTATGCTCTGTCTATGC-3', and the same conditions as GAPDH. Amplicons from the PCR were resolved by agarose gel electrophoresis and visualized by ethidium bromide staining.

### 3.4. Recombinant Baculovirus Expression

To analyze the protein products encoded by GP133, GP131 and GP129 regions of the GPCMV genome, proteins were expressed in recombinant baculovirus. Baculovirus shuttle plasmids were generated. Briefly, ORF coding sequences GP129, GP131 and GP133 were amplified from cDNA by PCR using the primers listed in Table 2. Amplicons were resolved by agarose gel electrophoresis and visualized by ethidium bromide staining. The purified amplicons were ligated into digested pAc-HLT-A-GFP vector (BD Biosciences, San Jose, CA, USA) at the C-terminus of the GFP-His tag following conventional cloning methods. The resultant clones encoding GP133, GP131 and GP129 were designated pKTS 824, pKTS 791 and pKTS 814, respectively. Recombinant baculoviruses were generated by co-transfecting Sf9 cells with aforementioned plasmids and BaculoGold® DNA (BD Biosciences) according to manufacturer’s instructions. 

### 3.5. Recombinant Antigenic GP129, GP131 and GP133 Fragment Expression

Antigenic domains for GP129, 131 and 133 were predicted using MacVector software (version 12.0) [35] by calculating antigenic index based on the results of Kyte-Doolittle hydrophilicity, surface probability, protein flexibility, and Chou-Fasman and Robson-Garnier secondary structure analyses. To screen for potential antigenic regions of GP129 that are recognized by the GPCMV guinea pig sera, three predicted antigenic fragments R1 (TYR_12_ through THR_50_), R2 (SER_79_ through ARG_129_) and R3 (ASN_146_ through LYS_179_) were expressed as GST fusion proteins. Briefly, R1, R2 and R3 coding sequences of GP129 were amplified from cDNA by PCR using the primers listed in Table 2. The purified amplicons were ligated into linearized pGEX-6P vector (GE Healthcare Biosciences, Pittsburgh, PA, USA) at the C−terminus of the GST following conventional cloning methods. The resultant clones encoding R1−GP129, R2-GP129, R3-GP129 were designated pKTS 828, 829 and 830, respectively. For GP131, the clones were designated as pKTS 818, 819, and 820 (domains R1, R2, and R3, respectively). For GP133, clones encoding R1-GP133 and R2-GP133 were designated as pKTS 831 and pKTS 832 respectively.

The aforementioned plasmids corresponded to these fusion proteins were sequenced, and then were transformed into MAX EFFICIENCY^®^ DH5α chemically competent cells (Invitrogen, Carlsbad, CA, USA) and screened on LB plates containing ampicillin (100 μg/mL). DH5 α cells harboring the above plasmids were selected to express GST fusion proteins under IPTG induction (0.5 mM). GST-fusion proteins were extracted and purified using Glutathione agarose beads (Pierce, Rockford, IL, USA) according to the manufacturer’s instructions.

### 3.6. Western Blot

Recombinant GP129, GP131 and GP133 proteins were characterized by western blot. Briefly, 80% confluent monolayer of Sf9 cells were infected with recombinant baculovirus and after 120 hours of infection, the cells were harvested and washed twice with PBS. Cellular proteins were extracted and recombinant GP129, GP131 and GP133 fusions proteins were purified using Ni-NTA agarose beads according to the manufacturer’s instruction (Qiagen). Either purified recombinant GP129, GP131 or GP133-expressing Sf9 cell lysates were resolved by SDS PAGE (Novex-10%–20% Tris-HCl, Life Technologies, Carlsbad, CA, USA) and electro blotted to PVDF membrane. Blots were blocked with 5% skim milk solution in PBS containing 0.1% Tween-20 for 2 hours. Blots were probed with anti−GFP monoclonal (1:3,000 dilution), anti-6-His monoclonal (1:2,000 dilution), anti-GST monoclonal (1:5,000 dilution), preimmune and post-infection sera from guinea pigs (1:200 dilution for most experiments as indicated in individual figure legends), pre-immune rabbit sera (both at 1:2,000 dilution), anti R3-GP129 guinea pig sera (1:2,000 dilution) or rabbit affinity-purified, GP131 anti−peptide antibody (concentration 1 µg/mL) for one hour, followed by incubation with either horseradish peroxidase (HRP)-conjugated anti-guinea pig (1:10,000 dilution, Sigma, St. Louis, MO, USA), HRP−conjugated anti-rabbit (1:2,000 dilution; Cell Signaling Technology, Danvers, MA, USA) or HRP-conjugated anti mouse (1:10,000; GE Healthcare Biosciences) secondary antibody. Anti−GPCMV gB moab IE321 was used at a 1:2,000 dilution. Binding of antibody was detected by adding enhanced chemiluminescent substrate (GE Healthcare Biosciences) for HRP and bands were visualized by exposing the blot to an X-ray film. 

### 3.7. Generation of Antibodies

GP131-specific anti-peptide antibodies were raised in rabbits (US Biological, Salem, MA, USA). A synthetic peptide, NH_2_-CYYPSTPIPKSFVKHVDTTRSLPE-COOH, was generated and conjugated with keyhole limpet hemocyanin (KLH). Rabbits were immunized with 200 µg of the conjugate and complete Freunds adjuvant (CFA), followed by three subsequent doses of the conjugate (100 µg) and incomplete Freunds adjuvant (IFA) at 21, 42 and 72 days following initial immunization. Serum was collected seven days after final immunization. GP131-specific anti-peptide antibodies were purified from the aforementioned rabbit serum by affinity column chromatography using cyanogen bromide-activated sepharose 4B gel coupled to NH_2_-CYYPSTPIPKSFVKHVDTTRSLPE-COOH.

R3-GP129 antibodies were raised in Strain 2 guinea pigs (University of Minnesota RAR facility, Minneapolis, MN, USA). GPCMV-seronegative guinea pigs were immunized with 50 µg of the purified GST_R3_GP129 and incomplete Freunds adjuvant (IFA), followed by three subsequent doses of the GST_R3_GP129 (30 µg) and incomplete Freunds adjuvant (IFA) at 21, 42 and 72 days following initial immunization. Sera were collected seven days after final immunization. 

GPCMV sera were generated by infecting seronegative Hartley strain guinea pigs (Elm Hill laboratories, Chelmsford, MA, USA) with salivary gland-adapted GPCMV. Infection was by subcutaneous route with a dose of 5 × 10^5^ pfu. Animals were monitored for seroconversion by ELISA assay and western blot, and preimmune and immune (post-infection) sera were used to probe GP129, 131 and 133 recombinant proteins at dilutions of 1:200 as described in individual figure legends.

## 4. Conclusions

These results confirm and extend the observations of Feierbach by identifying the GP129, 131 and 133 proteins in virus particles purified from GPCMV-infected cells [18]. There are potential limitations to our analyses. We performed these studies using strain 22122 of GPCMV from the ATCC. This strain is known to harbor two genomic variants of GPCMV, with the second variant containing a 1.6 kb genome deletion that lacks the GP129-133 locus. It is possible that our studies underestimated the presence of GP129-133 proteins by selecting for the deletion variant in cell culture in fibroblasts [16]. However, we used virus passaged only one time in GPL cells, and by PCR analysis we were able to confirm that the majority of the genome variants in cell culture were full-length, compatible with our finding of the GP129-133 proteins by mass spectrometry. Another potential limitation of these studies was the lack of full characterization of whether there were dense bodies co−purifying with virions in our purified virus. A more detailed characterization of GPCMV virions and dense bodies will be required in future studies to elucidate what differences exist in the proteome of these respective particles.

Using anti-peptide antisera (GP131) and an anti-GST fusion protein antibody (GP129), we were able to identify both the GP131 and GP129 proteins in purified virus particles by western blot. The GP131 was found to migrate as a doublet at ~20 and 22 kDa. The virion-associated GP129 migrated at ~25 kDa, compatible with its predicted potential to undergo more extensive glycosylation (Figure 5b). The GP129 protein also appeared to migrate as a double (at ~55 and ~65 kDa) in Sf9 cells infected with a recombinant baculovirus expressing GP129 as a GFP/6-His fusion, also compatible with differential glycosylated isoforms of the protein in insect cells. Experiments with glycosylation inhibitors will be required to examine this question in further detail. We were unable to identify GP133 in virions using an anti-GST antibody raised against a GP133-GST fusion protein (data not shown). However, the finding of GP133 by mass spectrometry analysis of viral particles provides assurance that this protein is expressed in the mature virion.

Using GST fusions and, for GP129, a recombinant baculovirus-expressed protein, we were able to identify antibody responses in serum from guinea pigs experimentally infected with GPCMV. We were unable to identify immunoreactivity of sera from GPCMV-infected animals with recombinant insect cell-expressed forms of GP131 and 133. Both the GP131 and GP133 baculovirus-expressed proteins were reactive with anti-6-His and anti-GFP antibodies at the predicted MWs of ~56 and ~49 kDa, respectively (data not shown), indicating that the recombinant baculovirus fusion protein was being expressed in insect cells. It may be that fusion of GFP and/or 6-His at the amino-terminus of these fusion proteins rendered relevant epitopes inaccessible. Our study was also limited by examination of a relatively small number of sera from infected animals.

In summary, GPCMV encodes homologs of HCMV PC complex proteins, GP129, 131, and 133, and these proteins are expressed and can be identified in virions by mass spectrometry. Furthermore, using recombinant GST and baculovirus expressed proteins, we observe that the GP129/131/133 constituents of the GPCMV PC appear to elicit an antibody response in the context of viral infection in animals. These observations reinforce interest in studying the GPCMV PC as a potential target of protective subunit vaccination in the GPCMV model of congenital infection. The precise protein-protein interactions in this complex remain to be elucidated. In a model of the protein-protein interactions that make up the gH/gL/UL128/UL130/UL131 complex, the HCMV gH protein is predicted to be inserted in the viral envelope, and interacts with gL through disulfide bond formation. Johnson *et al.* characterized the HCMV PC using a system of HCMV mutants lacking UL128-131, differentially reconstituted with non-replicating adenovirus vectors expressing gH, gL, UL128, UL130, and UL131 [4]. The assembly of a pentameric structure was further suggested by the observation of increased export of gH/gL complexes from the endoplasmic reticulum (ER) to the Golgi apparatus and cell surface when UL128, UL130, and UL131 were expressed with gH/gL [4]. Direct covalent disulfide bonds between HCMV gH/gL and UL130/UL131 were suggested by differential immunoprecipitation of heterodimers in reducing and non-reducing environments; direct non-covalent interactions between gH/UL130, gL/UL128, and UL128/UL130 were also observed. These complex interrelationships remain to be elucidated for the GPCMV PC. Evaluation of GPCMV neutralization following immunization with GPCMV pentameric complex constituents should help clarify which of these proteins are most important in elucidation of a protective immune response, and subunit vaccine studies in guinea pigs should help provide a useful proof-of-concept for the potential usefulness of a PC-based HCMV vaccine.
